# Editorial: Diagnostic and prognostic brain-based biomarkers in psychosis spectrum

**DOI:** 10.3389/fpsyt.2023.1332447

**Published:** 2023-11-22

**Authors:** Walid Yassin, Gil D. Hoftman, Sarah E. Bergen, Elisabetta C. del Re

**Affiliations:** ^1^Department of Psychiatry, Harvard Medical School, Boston, MA, United States; ^2^Department of Psychiatry, Semel Institute for Neuroscience and Human Behavior, University of California, Los Angeles, Los Angeles, CA, United States; ^3^Department of Medical Epidemiology and Biostatistics, Karolinska Institute, Stockholm, Sweden

**Keywords:** schizophrenia, biomarkers, psychosis, MRI, EEG, brain, tryptophan, kynurenine

The psychosis spectrum represents an umbrella of debilitating brain disorders affecting millions of lives. The common denominator is the presence of positive symptoms, characterizing schizophrenia (SZ), bipolar 1 disorder, as well as schizoaffective disorder, among others, with psychotic features. There is substantial neurobiological heterogeneity within these complex neuropsychiatric disorders, rendering their prognosis, diagnosis, and treatment difficult (1). There is an urgent and unmet need for biomarkers that are reliably, and consistently, capable of providing an objective layer of appraisal to aid clinical decisions (2). In this Research Topic we present findings highlighting the wide range of possibilities in the search for objective brain-based biomarkers, such as neuroimaging, electrophysiology and others that have the potential to identify disorder-specific brain signatures (3–6). A summary of this Research Topic is presented below.

A custom dense attention network (DAN) machine learning model was employed by Perellon-Alfonso et al. to discriminate between those with SZ and typically developing (TD) subjects using their electroencephalographic data. The model distinguished between the groups with high accuracy. The results from this work highlight the potential utility of interpretable machine learning algorithms as a promising tool in diagnosing SZ and other psychiatric disorders characterized by oscillatory abnormalities.

A different approach was undertaken by Takahashi et al.. Here, the authors focused on the insula, a region central to the default network and SZ pathophysiology (7). The authors demonstrated that the number of insular gyri in the anterior subdivision was higher bilaterally in the at risk for psychosis and SZ groups than the TD group. The SZ group in particular had a higher number of insular gyri in the left posterior subdivision. Abnormalities of insular gyri were associated with symptomatology and distinguished first episode psychosis vs. chronic probands.

The insula was also integral to a study by Zhou et al.. The team found a predisposition to an imbalance in the relative metabolism of kynurenine (KYN)/tryptophan (TRP) and KYN to gray matter volume (GMV) in SZ. The concentrations of kynurenic acid (KYNA) and the KYNA/KYN ratio were significantly higher in SZ compared to TD. KYN concentrations in SZ probands further negatively correlated with GMV in the left anterior cingulate belt while KYN/TRP negatively correlated with GMV of the left and right insula. The kynurenine pathway is the major metabolic pathway for tryptophan (TRP), an essential amino acid for the production of serotonin (8). The relevance here is the link between biochemical pathways important in neurotransmission through metabolism of tryptophan and the cortex in SZ (9).

The perspective article by Stuke, on muscarinic acetylcholine receptors contributes important information to the search for brain-based biomarkers in psychiatry. Some clinical studies have shown that agonists at muscarinic acetylcholine receptors can ameliorate SZ symptoms. Research in this field is promising and might be an initial step to develop novel psychotropic medicines targeting acetylcholine receptors to accompany the traditional antidopaminergic medications currently available. Supportive evidence has indeed shown that decreased cortical muscarinic receptors might identify specific groups of probands affected by Scarr et al. (10).

Taken together, the perspective and collection of findings discussed above demonstrate the importance of investigating brain-based biomarkers because of their potential merit in identifying neurobiological signatures that may contribute to diagnosis, prognosis, and treatment of psychosis. It is also clear that different approaches, like the ones highlighted in this Research Topic, are critically needed because of the heterogeneous nature of the psychosis spectrum.

Future endeavors might entail combining different approaches and assessments in the search for individualized diagnoses, prognoses and especially prevention. Here, artificial intelligence methods might be key in making sense of the heterogenous illness presentations.

## Author contributions

WY: Conceptualization, Writing – original draft, Writing – review & editing. GH: Writing – original draft, Writing – review & editing. SB: Writing – review & editing. ECdR: Conceptualization, Writing – original draft, Writing – review & editing.
